# Identification and Classification of Facial Familiarity in Directed Lying: An ERP Study

**DOI:** 10.1371/journal.pone.0031250

**Published:** 2012-02-21

**Authors:** Delin Sun, Chetwyn C. H. Chan, Tatia M. C. Lee

**Affiliations:** 1 Laboratory of Neuropsychology, The University of Hong Kong, Hong Kong, China; 2 Laboratory of Cognitive Affective Neuroscience, The University of Hong Kong, Hong Kong, China; 3 Applied Cognitive Neuroscience Laboratory, Department of Rehabilitation Sciences, The Hong Kong Polytechnic University, Hong Kong, China; 4 The State Key Laboratory of Brain and Cognitive Sciences, The University of Hong Kong, Hong Kong, China; Cuban Neuroscience Center, Cuba

## Abstract

Recognizing familiar faces is essential to social functioning, but little is known about how people identify human faces and classify them in terms of familiarity. Face identification involves discriminating familiar faces from unfamiliar faces, whereas face classification involves making an intentional decision to classify faces as “familiar” or “unfamiliar.” This study used a directed-lying task to explore the differentiation between identification and classification processes involved in the recognition of familiar faces. To explore this issue, the participants in this study were shown familiar and unfamiliar faces. They responded to these faces (i.e., as familiar or unfamiliar) in accordance with the instructions they were given (i.e., to lie or to tell the truth) while their EEG activity was recorded. Familiar faces (regardless of lying vs. truth) elicited significantly less negative-going N400f in the middle and right parietal and temporal regions than unfamiliar faces. Regardless of their actual familiarity, the faces that the participants classified as “familiar” elicited more negative-going N400f in the central and right temporal regions than those classified as “unfamiliar.” The P600 was related primarily with the facial identification process. Familiar faces (regardless of lying vs. truth) elicited more positive-going P600f in the middle parietal and middle occipital regions. The results suggest that N400f and P600f play different roles in the processes involved in facial recognition. The N400f appears to be associated with both the identification (judgment of familiarity) and classification of faces, while it is likely that the P600f is only associated with the identification process (recollection of facial information). Future studies should use different experimental paradigms to validate the generalizability of the results of this study.

## Introduction

Recognizing familiar faces is crucial to social interaction, one of the basic abilities of human beings [1], [2], [3]. People act very differently if they categorize someone as a friend or a stranger. The ability to differentiate a friend from a stranger and to make appropriate responses in terms of greetings (e.g., addressing by name), facial expressions (e.g., smiling), and body gestures (e.g., hand shaking) have serious social consequences. However, people sometimes deliberately pretend not to recognize faces for their own benefit. For instance, a debtor might deny recognizing a loan shark in a face-to-face encounter in order to avoid the demand to pay up, or a swindler may sidle up to a stranger, pretending to recognize a relative, to ask for money. These examples suggest that processing familiar faces may involve two dissociable processes: identification and classification. The identification process probably involves perceiving facial features and relating the face to other semantic information, such as names, relationships, and events. The classification process probably involves the intention of recognizing a face, which may be outcome driven.

Reviewing the research on face recognition, there is a unitary model that stipulates that people always try to recognize faces accurately (i.e., to recognize familiar faces as “familiar” and unfamiliar faces as “unfamiliar”) [4], [5], [6]. A few recent fMRI studies have investigated the effect of the deliberate manipulation of facial recognition [7], [8]. These studies focused on the neural processes involved in the manipulation of intention and used recognition of faces as an outcome measure, but they did not address the separate processes of identification and classification. Furthermore, the poor temporal resolution of fMRI prevented these researchers from finely differentiating these neural processes.

The ERP methodology offers a high temporal resolution that helps uncovered at least two processes associated with processing familiar faces [9], [10]. Eimer [9] found that compared to unfamiliar faces, familiar faces elicit an enhanced N400f (300–450 ms post stimulus) and P600f (450–650 ms). Both the N400f and the P600f were widely distributed over the scalp and peaked in the central-parietal region. These two components were not observed when participants saw inverted faces, which have been known to disrupt face recognition [11], [12]. They were also absent in prosopagnostic patients, who suffer from semantic memory dysfunction, further suggesting that N400f and P600f are important in face recognition [13].

The question then becomes “What roles do N400f and P600f play in face recognition?” There are at least two theories. First, the earlier N400f could be the identification of the actual identity of the face, and the later P600f could be the intentional classification of the face into either the “familiar” or “unfamiliar” category. This theory is not terribly promising, since a previous study found that the P600f was not necessarily a consequential process of the N400f. Eimer [10] found that when participants had to respond to a target character inside a string presented in front of the facial stimulus, the letter strings directed attention away from the face, after which the N400f disappeared, but the P600f remained, for familiar faces. One interpretation of this finding is that the processes responsible for the N400f are not triggered automatically in response to the presence of a familiar face in the visual field; instead, they depend on attentional processing. The fact that the P600f remained suggests that it is independent from attentional processing. In line with this thought, the N400f might be the result of a top-down cognitive control process, while the P600f may reflect the more basic processing of the characteristics of the stimuli (facial features and semantic content). A second possible theory is that the P600f reflects the identification process, which includes the perception of facial features and relating the features to the semantic content and leads to recognition. The N400f may represent the classification of facial familiarity, which is a top-down intention to manipulate incoming information.

This study investigates the roles of N400f and P600f in facial recognition by manipulating classification intention using a directed-lying paradigm. Participants were asked to respond to the familiarity of facial stimuli according to visual cues that prompted them to make either a truthful (i.e., congruent) or deceptive (i.e., incongruent) response. It was hypothesized that the directed-lying task would elicit two event-related components: one around 400 ms post stimulus (probably the N400f) elicited the strongest signal in the central-parietal region and another around 600 ms post stimulus (probably the P600f), also elicited the strongest signal in the central-parietal region. Furthermore, it was hypothesized that the N400f would be modulated by congruency and incongruency (lying) and the P600f would be modulated by (actual) familiarity and unfamiliarity.

## Results

### Behavioral findings

Measured by mean reaction times, there were no significant effect of identification (*F*<1), but there were significant effects of classification (*F*(1, 14) = 1.64, *p* = .022) and the interaction between identification and classification (*F*(1, 14) = 3.37, *p* = .009).

Measured by accuracy rates, there was a significant main effect of identification (*F*(1, 14) = 5.66, *p* = .003) but not of classification (*F*<1). The interaction between identification and classification was also significant (*F*(1, 14) = 4.95, *p* = .004). In general, the participants identified familiar faces (97.2±3.0%) more accurately than unfamiliar faces (95.3±4.1%). When the participants saw familiar faces, they were more accurate when they were instructed to tell the truth (97.9±2.5%) than when they were told to lie (96.6±3.4%; i.e., incongruent), *t*(14) = 2.23, *p* = .004. There were no other significant differences in the between-condition comparisons.

### ERP results

SPM analyses showed significant main effects of identification on the amplitudes of ERP in two time ranges. In general, the amplitudes elicited by familiar faces were significantly more positive-going than amplitudes from unfamiliar faces. The first component was found before 400 ms post stimulus, with maximal significance identified at 319 ms. The second component was found after 400 ms post stimulus, with maximal significance identified at 501 ms. The maximal significance of both components appeared to come mainly from channels in the middle and right parietal regions and from the middle and right temporal regions. There was less amplitude in the middle occipital region (Table 1).

**Table 1:** Main and interaction effects of identification and classification revealed by repeated-measures ANOVA using SPM (Significance level *p*<.001, uncorrected. Extent threshold *k*>200 voxels).

*t(ms)*	*side* #	*area* $	*channel* *	*k*	*T*	*p(unc)*
**Main effect of identification**						
***(FF+FU)>(UF+UU)***						
284	R	T	127	670	3.41	0.001
286	R	FT	112	670	3.34	0.001
319	M	P	67	23078	4.32	<0.001
326	M	P	94	23078	4.16	<0.001
327	R	PT	121	23078	4.23	<0.001
371	R	T	127	1762	3.53	<0.001
371	R	PT	121	1762	3.44	0.001
372	R	FT	112	1762	3.48	<0.001
501	M	P	72	364264	6.94	<0.001
501	M	O	70	364264	6.81	<0.001
541	M	P	67	364264	6.59	<0.001
***(FF+FU)<(UF+UU)***						
No significance						
**Main effect of classification**						
***(FF+UF)>(FU+UU)***						
No significance						
***(FF+F)<(FU+UU)***						
280	M	C	51	34450	4.36	<0.001
284	M	C	90	34450	4.36	<0.001
287	R	T	127	34450	4.19	<0.001
761	M	O	45	2025	3.74	<0.001
761	M	O	69	2025	3.65	<0.001
762	L	OT	22	2025	3.75	<0.001
763	R	FT	112	460	3.48	<0.001
764	R	T	128	2556	3.62	<0.001
765	R	PT	121	2556	3.63	<0.001
**Interaction between stimuli and response**						
***(FF−FU)>(UF−UU)***						
No significance						
***(FF−FU)<(UF−UU)***						
No significance						

# denotes the transverse positions: i.e., left (L), middle (M), and right (R).

$ denotes the area the channel is located in: i.e., frontal-temporal (FT), central (C), parietal (P), parietal-temporal (PT), temporal (T), occipital (O), and occipital-temporal (OT).

*denotes the nearest suprathreshold channel.

*k* = cluster size (number of voxels showing significant differences; each voxel size is 2.13 mm×2.69 mm×1 ms), *T* = peak value measured within the cluster, FF = familiar faces classified as “familiar,” FU = familiar faces classified as “unfamiliar,” UF = unfamiliar faces classified as “familiar,” and UU = unfamiliar faces classified as “unfamiliar.”

**Figure 1:**
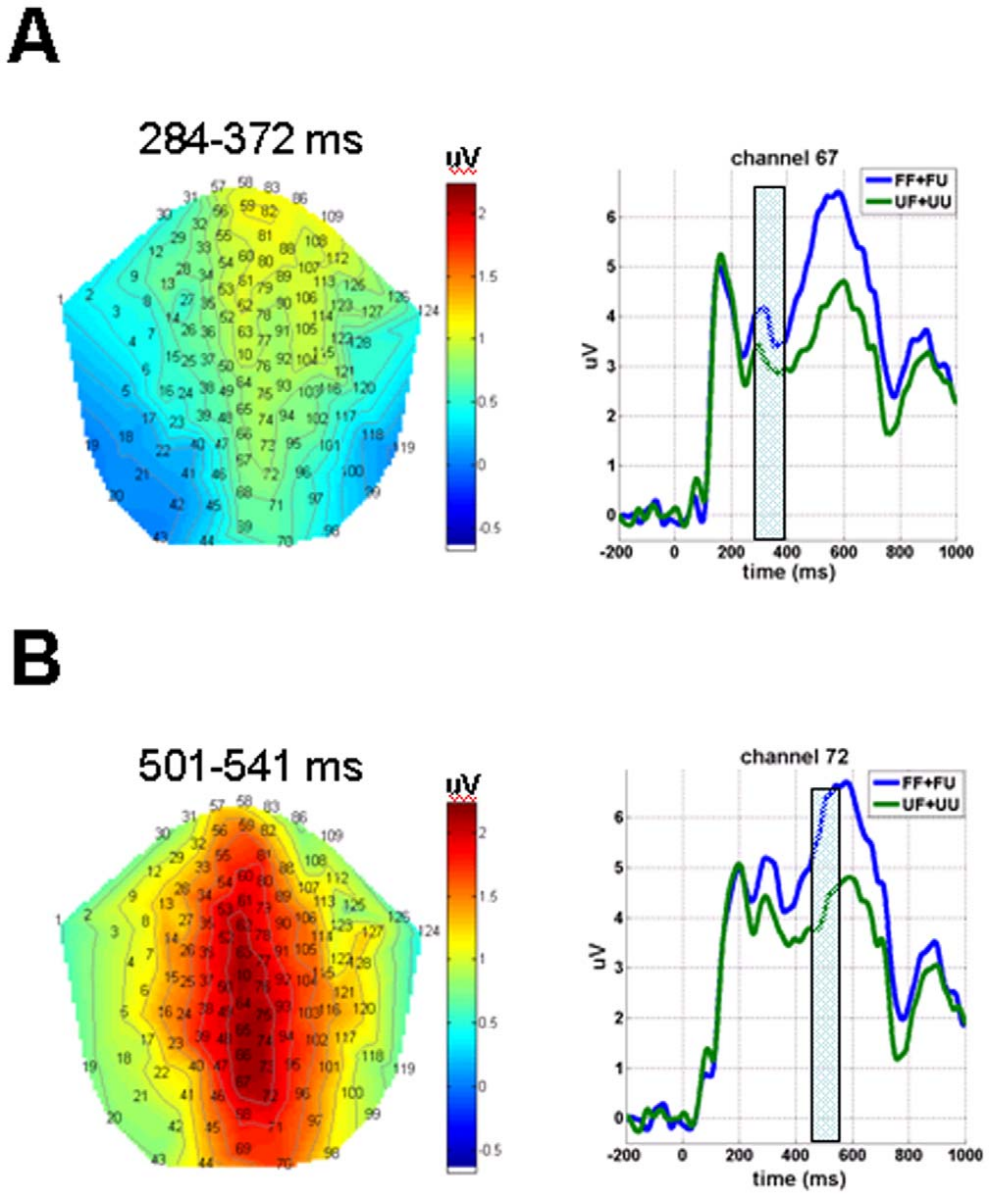
ERP waveforms at representative channels indicating the main effect of the identification of facial familiarity. Familiar faces elicited significantly more positive-going amplitudes than unfamiliar faces at two time windows: (A) From 284 to 372 ms (N400f) post stimulus in the middle parietal (represented by channel 67) and right parietal regions; (B) From 501 to 541 ms (P600f) post-stimulus in the middle parietal (represented by channel 72) and occipital regions. The shadowed bars cover the time windows indicated above. Fam = familiar faces, Unfam = unfamiliar faces, “Fam” = responded as familiar faces, and “Unfam” = responded as unfamiliar faces.

It is likely that the maximum significance at 319 ms (from 284 to 372 ms) represents the N400f. The main channels elicited less negative-going N400f (i.e., familiar>unfamiliar) in the middle (67 and 94) and right (121) parietal regions (*p*<.001; *k* = 23,078 voxels; Table 1, Figure 1A). Other channels showing a similar pattern included those in the right frontal-temporal, temporal, and temporal-parietal regions (112 and 127; *p* = .001 to <.001; k = 670 or 1,762 voxels). It is likely that the maximum significance at 501 ms (from 501 to 541 ms) represents the P600f. The channels elicited more positive-going P600f (i.e., familiar>unfamiliar) in the middle parietal (67 and 72) and middle occipital (70) regions (*p*<.001; *k* = 364,264 voxels; Figure 1B).

**Figure 2:**
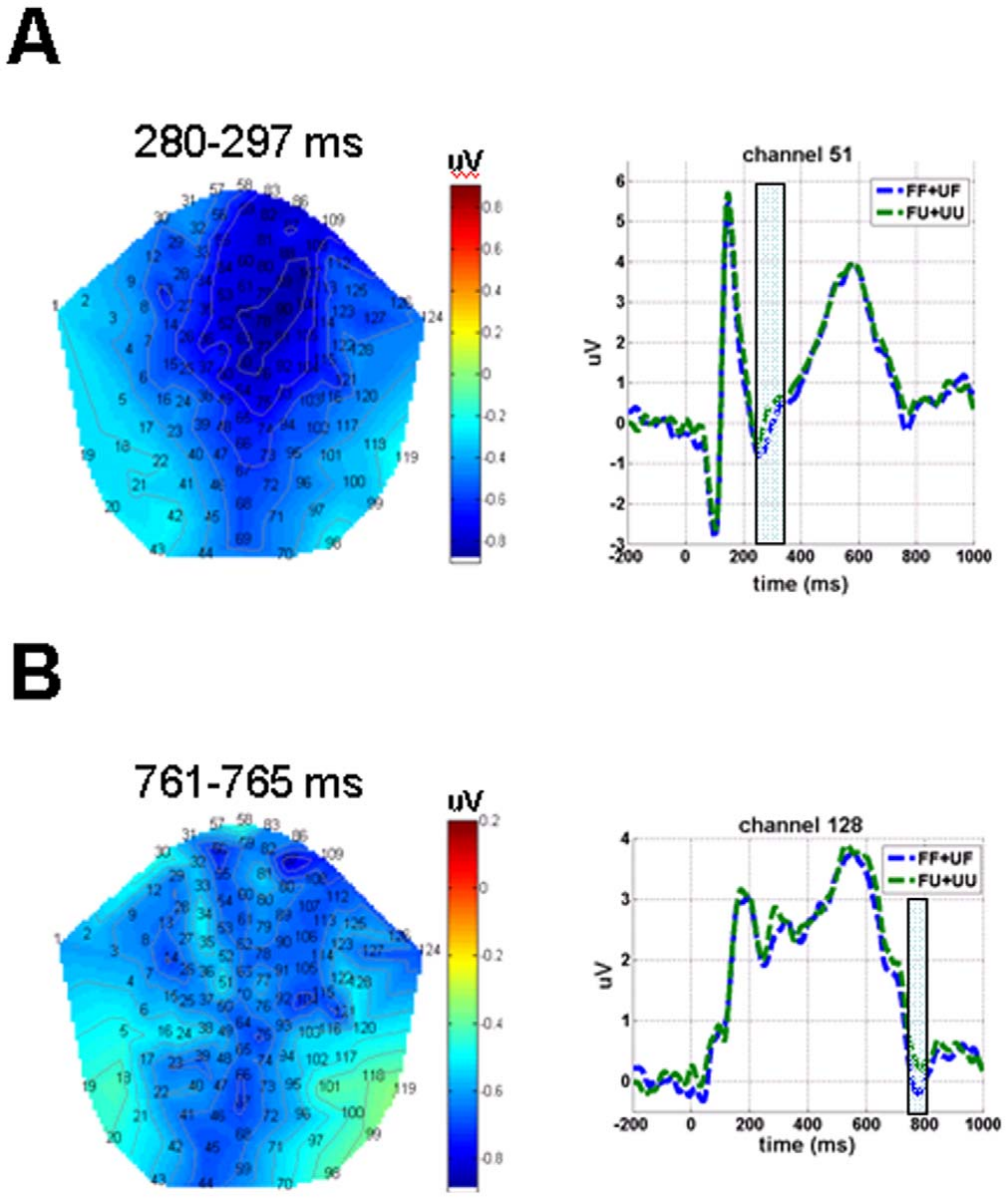
ERP waveforms at representative channels indicating the main effect of classification of facial familiarity. Faces classified as “familiar” elicited significantly more negative-going amplitudes than faces classified as “unfamiliar” at two time windows: (A) From 280 to 297 ms (N400f) post stimulus in the middle central (represented by channel 51) and right temporal regions; (B) From 761 to 765 ms (P600f) post stimulus in the right temporal (represented by channel 128), middle occipital and left temporal-occipital regions. The shadowed bars cover the time windows indicated above. Fam = familiar faces, Unfam = unfamiliar faces, “Fam” = responded as familiar faces, and “Unfam” = responded as unfamiliar faces.

There was a significant main effect of classification in two time ranges. Unlike the identification effect, the amplitudes elicited by classifying faces as “familiar” were significantly less positive-going than those elicited by classifying faces as “unfamiliar.” The first component, which was likely to be a more negative-going N400f (i.e., “familiar”<“unfamiliar”), was found to be maximally significant around 280 ms post stimulus and was elicited at channels in the middle central region (51 and 90) and the right temporal (127) regions (*p*<.001; *k* = 34,450 voxels; Table 1, Figure 2A). The second component associated with classification was between 761 and 765 ms post stimulus and was elicited in channels located in the right temporal (112, 121, and 128), middle occipital (45 and 69), and left temporal-occipital (22) regions (*p*<.001; *k* = 2,023 to 2,556 voxels, except for channel 112, which was 460 voxels; Figure 2B).

SPM analysis did not show significant interactions between the identification and classification effects on the ERP amplitudes at the significance level of *p*<.001.

Conventional analyses based on the mean amplitudes extracted from the selected channels within the predetermined time windows of N400f and P600f revealed findings similar to those found using the SPM method. There were significant main effects of both identification (*F*(1, 14) = 11.19, *p* = .005) and classification (*F*(1, 14) = 9.22, *p* = .009) for the N400f. However, the conventional analysis revealed a time window from 320 to 400 ms—somewhat later than the time points of peak significance signaled by the SPM (284 to 372 ms). The interactions between the identification, classification, and site effects were not significant (*F*(3,42) = 1.33 to 2.45, *p*>.05).

Familiar faces elicited significantly less-negative going N400f than unfamiliar faces at the midline sites (i.e., familiar>unfamiliar); faces classified as “familiar” (regardless of truth/lying) elicited more negative-going amplitudes than those classified as “unfamiliar” at the midline sites. Only the effect of identification (*F*(1, 14) = 26.15, *p*<.001) was significant in modulating the P600f amplitudes. All of the other main (*F*(1, 14) = 2.14 to 3.01, *p*>.05) and interaction (*F*(3, 42) = <1 to 1.13 *p*>.05) effects were not statistically significant.

Familiar faces elicited more positive-going P600f over the midline sites than unfamiliar faces. The main effect of classification found by the SPM at the right temporal, middle occipital, and left temporal-occipital regions from 761 to 765 ms was beyond the time window of the P600f (i.e., 500 to 600 ms) and was not found in the conventional analysis.

## Discussion

This study used a directed-lying paradigm to explore the possible differentiation between identification and classification processes involved in facial recognition. The directed-lying paradigm made it possible to measure honest and deceptive classification responses for familiar and unfamiliar faces. In general, the participants were more accurate at identifying familiar faces than unfamiliar faces. Results based on the two a priori markers for facial familiarity showed that the N400f occurred with both identification and classification, while P600f only appeared with identification. Regardless of whether they had to lie or tell the truth, the participants had more positive-going N400f and P600f in the middle and right parietal regions when they were identifying familiar faces compared to when they were identifying unfamiliar faces. Classifying faces as “familiar” (under both truth and lie conditions) elicited more negative-going N400f in the central and right temporal regions. Both conventional and SPM analysis gave convergent evidence of these observations.

The results showed that the N400f was associated with both the identification and classification processes in facial recognition. The difference is that in the identification process, familiar faces were found to elicit less negative-going N400f than unfamiliar faces. In contrast, in the classification process, faces classified as familiar elicited more negative-going N400f than those classified as unfamiliar. This suggests that it is likely that, despite sharing the same N400f, the two steps in facial recognition involve two discrepant cognitive processes. Previous studies have consistently found that the N400f is involved in discriminating familiar faces from unfamiliar ones [14], [15], [16]. Previous researchers have also related the N400f to high-level attentional processing [10]. They found that familiar faces elicited more negativity than unfamiliar faces at Cz and Pz; however, such effects were diminished when attention was directed to another demanding task. It is plausible that the N400f also reflects top-down control in the processing of familiar faces.

In this study, regardless of whether the participants were telling the truth or lying, faces classified as “familiar” elicited more negative-going N400f than those classified as “unfamiliar.” These results were interesting because lying did not seem to affect the N400f. In other words, classification could be determined by the intention of categorizing faces as familiar or unfamiliar rather than by the possible regulatory processes of lying.

Moreover, there were different polarity effects for identification (i.e., familiar>unfamiliar) and classification (i.e., “familiar”<“unfamiliar”). Compare this to the findings of previous studies that found N400f amplitude differences: familiar faces classified as familiar elicited more negative-going amplitudes than unfamiliar faces classified as unfamiliar [9], [10]. This suggests that the N400f can also be attributed to an intended classification of the facial stimuli rather than to the mere identification of familiarity based on facial features.

The P600f was associated only with the identification of faces, not with lying. Familiar faces elicited a more positive-going component than unfamiliar faces in the middle parietal and occipital regions. This identification process seems to be independent from the classification process since the interaction was not significant. Our findings are consistent with a previous study that reported that the P600f component was related with more positive amplitudes for familiar faces than for unfamiliar faces at the Pz channel, regardless of whether attention was directed to another demanding task or not [10]. The association of face identification with the P600f is somewhat counter-intuitive. One would expect the perception of facial features to be a prerequisite for identification, which precedes classification, but the results of this study suggest that some sub-process of identification (reflected by the P600f) takes place even after classification (reflected by the N400f). Rugg and Curran offered a plausible explanation for the temporally split phenomena underlying face identification [17]. According to their findings, the recognition of an object involves a dual process: a judgment on familiarity followed by a recollection of the information associated with the object. The identification sub-process, as reflected by the P600f in this study, suggests that the participants probably accessed the information related to the familiar faces. This proposition is supported by previous studies which related the P600f to the processing of the demographic characteristics (e.g., name and occupation) of known persons or the visual characteristics of known faces [9], [10], [13]. The recollection of information about familiar faces is likely to be automatic as the participants were not instructed recollect such information in the task used in this study.

In the directed-lying task used in the study, the intention to lie or tell the truth was prompted before the presentation of a face. The appearance of the face would have enabled the participants to extract the facial features embedded in the stimuli (associated with N170). Setting the intention to lie as part of the classification process appears to occur at around 280 ms (denoted by N400f). Our results also suggest that attention would be allocated as part of the identification process at round 300 ms. The extraction of semantic information about faces, which is independent of the decision to lie (classification), is likely to occur at around 500 ms.

An interesting finding in this study is the late onset of the classification process, elicited around 760 ms in the bilateral temporal to occipital regions. Temporally, this signal is distinct from the N400f and P600f components. Other studies have reported a face-specific negative component (N700) elicited about 700 ms post stimulus from intracranial electrodes placed on the cortical surface of the ventral and lateral brain regions [17], [18]. The N700 was found to be related to semantic priming in a task involving learning and identifying face names [17], perhaps reflecting a top-down process. However, the contribution of this component in the processing of facial familiarity is beyond the scope of this study and should be investigated in further studies.

Our findings support the differential roles of identification and classification processes in the recognition of familiar faces. The N400f appears to be linked with both identification and classification, while the P600f appears to be primarily linked with identification. The dual identification processes revealed in this study are likely to involve an earlier judgment of familiarity and a later recollection of information related to familiar faces. Using electrophysiological measures together with brain imaging could further differentiate the role of these two components.

This study has a few limitations. The faces used were of personal acquaintances, but previous studies have shown that learned familiarity [13], [19] and celebrity faces [20] evoke different processes. Therefore, caution should be taken when generalizing the current findings to female adults and those with different demographic characteristics to the participants in this study. The results may also not be generalizable to other types of familiarity. More studies on the neural processing of face identification and classification are still needed.

This study also has implications for lie detection. However, there are at least two things that need to be done first. First, this paradigm needs to be tested on more samples to obtain a consistent standard for lie detection. Second, the measures should be adjusted to the special neural characteristics of each person so that the method is able to detect lies better in individual cases.

## Materials and Methods

### Participants

Fifteen healthy Chinese males aged between 25 to 40 years (mean = 29.8; SD = 4.0) and with an average of 16.2 years of education (SD = 2.9) were recruited from the community. Male participants were recruited to prevent possible gender differences in facial recognition [21], [22] and directed-lying behaviors [23], [24], [25]. All of the participants (a) were right handed as assessed by the Edinburgh Handedness Inventory [26], (b) possessed normal or corrected-to-normal vision, (c) reported no history of neurological or mental disorders, and (d) gave their written informed consent to participate in the study. The study was conducted under the approval of the Institutional Review Board of The University of Hong Kong/Hospital Authority Hong Kong West Cluster and Hong Kong Polytechnic University.

### Materials

There were two sets of face stimuli: (1) 30 personally familiar faces of males taken from photographs of the participants' friends from the local community and (2) 30 faces of male strangers, which were photos of Chinese people unknown to the participants. All of the familiar and unfamiliar stimuli had neutral facial expressions and were age matched. To control for differences in color tones, all of the pictures were transformed into grayscale. The luminance, contrast, and resolution of the photos were adjusted to approach equivalence using Adobe Photoshop (San Jose, CA).

### Experimental Task

**Figure 3:**
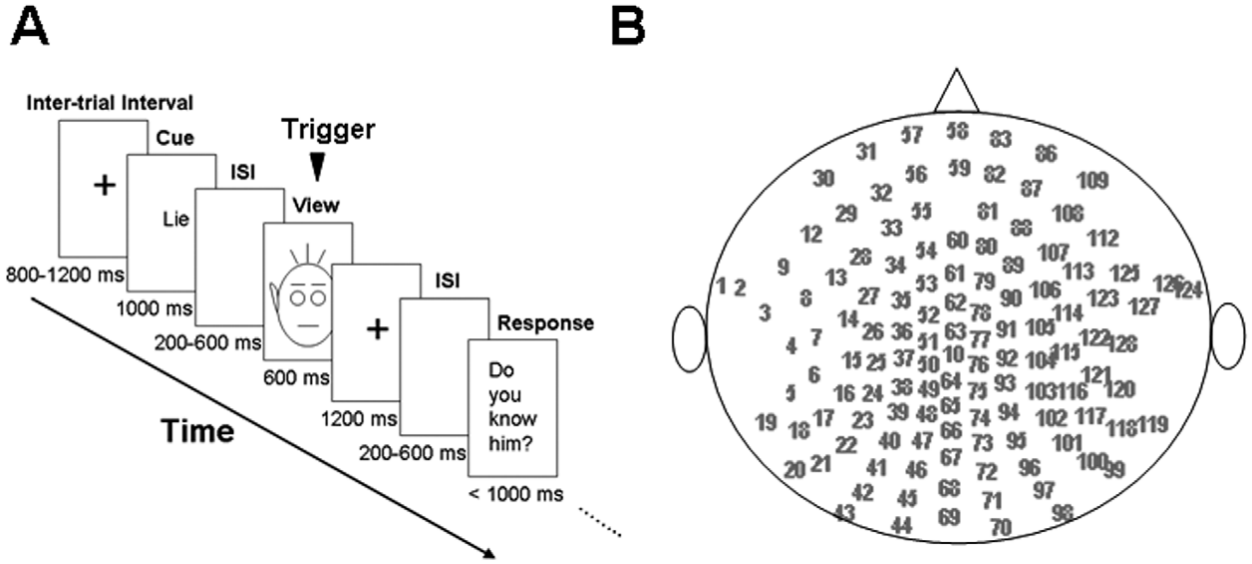
Task paradigm and 2D map of channels. (A) Schematic paradigm of the directed-lying task. In each trial, after a randomized inter-trial interval of 800 to 1,200 ms, a condition cue of either “Lie” or “Truth” was presented on the screen for 1,000 ms. This was followed by a randomized 200 to 600 ms blank screen inter-stimulus interval (ISI), after which a face appeared on the screen for 600 ms. A fixation cross was presented on the screen for 1,200 ms to allow time for the participants to prepare their response. Finally, after a randomized 200 to 600 ms blank ISI, the question “Do you know him?” appeared on the screen to prompt a response. Participants were asked to respond within 1,000 ms in accordance with the lie/truth cue presented previously. (B) 2D map of the channels distributed on the scalp. The top denotes the frontal side and the left denotes the left side.

This study's design was based on Lee and colleagues' design [8]. After a randomized inter-trial interval ranging from 800 to 1,200 ms, a condition cue of “Lie” (incongruent response) or “Truth” (congruent response) was presented on the screen for 1,000 ms (Fig. 3A). This was followed by a blank screen that lasted for a randomized period of 200 to 600 ms. The face stimulus was then presented for 600 ms. A fixation cross then appeared on the screen for 1,200 ms to give the participants time to prepare a response. Then, after a randomized 200 ms to 600 ms blank inter-stimulus interval (ISI), the question “Do you know him?” appeared on the screen to prompt the participants to make a response. The participants had to respond by indicating whether the face shown was “Familiar” or “Unfamiliar” by pressing the designated keys on a keypad using the left or right index finger.

The participants were told to respond as quickly and accurately as possible. The keys for “Familiar” and “Unfamiliar” were counterbalanced among the participants. The two main effects manipulated were identification (familiar vs. unfamiliar faces) and congruence (truth vs. lying). The 2×2 factorial design gave four conditions: (1) familiar faces classified as “familiar” (a “Truth” cue leading to a congruent response); (2) familiar faces classified as “unfamiliar” (a “Lie” cue leading to an incongruent response); (3) unfamiliar faces classified as “familiar” (“Lie”/incongruent); and (4) unfamiliar faces classified as “unfamiliar” (“Truth”/congruent). During the formal experiment, each of the 60 faces (30 familiar and 30 unfamiliar) was presented 8 times, i.e. half for truthful responses and half for deceptive responses, giving a total of 480 trials in the task. The order of trials was randomized into 8 blocks. Completing one block took about 5 minutes, and the participants had 30-second breaks between the blocks.

### Procedure

Prior to the experimental task, each participant completed familiarity and valence calibrations of the facial stimuli used in the experimental task. After seeing a face, the participant was required to give the name of the person corresponding to the face and to assign a rating of the extent of the face's familiarity and valence, from 1 (lowest) to 9 (highest). Stimuli that produced correct name responses, familiarity ratings above 5 (the middle score), and valence ratings of 5 (to control for possible valence effects) were included in the experimental task as familiar faces. Faces were selected as the unfamiliar face stimuli if the participants (1) could not name the person, (2) assigned a 0 familiarity rating, or (3) assigned a valence rating of 1 to 4. This maximized the difference in familiarity between the familiar and unfamiliar faces.

The directed-lying task was explained to the participants as a lie-detection game in which they were required to lie as genuinely as possible so as to deceive the computer. They were instructed to pay attention to the visual cue of “Truth” or “Lie” (all materials were written in Chinese) before the presentation of the stimulus. For example, a “Truth” cue would mean responding “familiar” when seeing a familiar face; a “Lie” cue would mean responding “unfamiliar” to a familiar face.

The participants practiced with a few trials until they expressed their readiness to engage in the experimental task. During the experiment, each participant was seated about 0.5 m in front of a computer screen in an electromagnetically shielded room. All of the visual stimuli were presented within 10 degrees of the visual angle to control eye movement.

### Behavioral data analysis

The variables under study were response time and accuracy with a 2 (familiar vs. unfamiliar faces)×2 (lie vs. truth) repeated-measures ANOVA.

### ERP recording and analysis

Electroencephalogram (EEG) data was captured over the scalp by a 128-channel fabric cap (Neuroscan) embedded with Ag-AgCl electrodes. All channel recordings were referenced to a computed average of the left and right mastoids. Channel impedances were kept below 5 kΩ. The electrical signals were amplified by a gain of 1,000 with a band pass from .01 to 200 Hz.

The preprocessing of EEG data was conducted with Scan 4.3 (Neuroscan). The raw signals were filtered off-line with a zero phase-shift digital filter and a 0.1 to 30 Hz band pass. Eye blink artifacts were mathematically corrected [27], and signals exceeding ±100 µV were automatically discarded. The epochs extracted covered −200 to 1,000 ms of each trial, with time zero set at the time when the facial stimulus was presented. Epochs of each of the 2×2 conditions were averaged for each participant.

Averaged epochs (i.e., files ending with “.avg”) for each participant were converted into SPM8 (Wellcome Trust Centre for Neuroimaging, UCL) file format on Matlab (The MathWorks, Inc.). The first step was to generate scalp maps per time frame using the 2D sensor layout [28], [29]. The output dimension of an interpolated scalp map was 64 pixels in each of the *x* and *y* directions. That is, the standard Neuroscan 128-channel locations (for ensuring smoothness) were projected onto a 64×64 pixel sensor space equivalent to 136.32 mm×172.16 mm. The second step was to stack scalp maps over peristimulus time. A total of 1201 scalp maps were constructed from epoch based on the 1,000 Hz sampling rate. This generated the 3D (64×64×1201 voxels) data volume for computation. Different from functional brain mapping, a voxel in here was defined as 2.13 mm (space)×2.69 mm (space)×1 ms (time). The ERP amplitudes captured at each channel and time point were fit to the voxels by linear interpolation and Gaussian smoothing procedures (at FWHM correction 8∶8∶8) [30], [31].

The SPM8 used a general linear model (GLM) to analyze the main and interaction effects on these images. Because the a priori hypotheses set for the present study were about the N400f and P600f, signals captured earlier than P1 (up to 80 ms post stimulus, which were primarily due to visual information processing [18]) and later than 980 ms were excluded from the analysis. Similar to the main effects used for the behavioral data, a 2×2 repeated-measures ANOVA tested the identification and classification effects on the two ERP components. Significance thresholds were set at *p*<.001 (uncorrected), and the extent threshold was set at *k*>200 voxels; it was set conservatively to prevent false positives.

The SPM method results were verified against conventional repeated-measures ANOVA model analyses of the amplitudes of N400f and P600f captured at selected sites on the scalp. The sites selected for the analyses were based on the sites used in previous studies [9], [10] over the midline central-parietal regions. Four sites along the midline were selected: Fz, Cz, Pz, and Oz; these sites were equivalent to channels 60, 10, 66, and 69, respectively (Fig. 3B). The time window defined for the N400f (320 to 400 ms) and P600f (500 to 600 ms) components were based on Eimer's studies [9], [10]. The mean amplitude of each component was tested with a 2 (familiar vs. unfamiliar face)×2 (lie vs. truth)×4 (sites: Fz, Cz, Pz, and Oz) repeated-measures ANOVA.
